# Assessing the HIV care continuum among transgender women during 11 years of follow‐up: results from the Netherlands’ ATHENA observational cohort

**DOI:** 10.1002/jia2.26317

**Published:** 2024-08-08

**Authors:** Vita W. Jongen, Ceranza Daans, Ard van Sighem, Maarten Schim van der Loeff, Kris Hage, Camiel Welling, Alex von Vaupel‐Klein, Martin den Heijer, Edgar J. G. Peters, Marc van der Valk, Peter Reiss, Maria Prins, Elske Hoornenborg, the ATHENA observational HIV cohort

**Affiliations:** ^1^ Department of Infectious Diseases Public Health Service Amsterdam Amsterdam The Netherlands; ^2^ Stichting hiv monitoring Amsterdam The Netherlands; ^3^ Amsterdam UMC University of Amsterdam, Internal Medicine Amsterdam The Netherlands; ^4^ Amsterdam Institute for Infection and Immunity (AII) Amsterdam The Netherlands; ^5^ Amsterdam Public Health Research Institute (APH) Amsterdam The Netherlands; ^6^ Trans United Europe Amsterdam The Netherlands; ^7^ Amsterdam UMC Vrije Universiteit Amsterdam, Internal Medicine Amsterdam The Netherlands; ^8^ Center of Expertise on Gender Dysphoria, Amsterdam UMC VU University Medical Center Amsterdam The Netherlands; ^9^ Amsterdam UMC University of Amsterdam, Global Health Amsterdam The Netherlands; ^10^ Amsterdam Institute for Global Health and Development Amsterdam The Netherlands

**Keywords:** transgender women, transgender people, HIV, HIV acquisitions, HIV epidemiology, HIV care continuum

## Abstract

**Introduction:**

Transgender women are at increased risk of acquiring HIV. Earlier studies reported lower retention in HIV care, antiretroviral therapy uptake, adherence and viral suppression. We assessed the stages of the HIV care continuum of transgender women in the Netherlands over an 11‐year period. In addition, we assessed new HIV diagnoses and late presentation, as well as disengagement from care, between 2011 and 2021.

**Methods:**

Using data from the Dutch national ATHENA cohort, we separately assessed viral suppression, as well as time to achieving viral suppression, among transgender women for each year between 2011 and 2021. We also assessed trends in new HIV diagnoses and late presentation (CD4 count of <350 cells/µl and/or AIDS at diagnosis), and disengagement from care.

**Results:**

Between 2011 and 2021, a total of 260 transgender women attended at least one HIV clinical visit. Across all years, <90% of transgender women were virally suppressed (207/239 [87%] in 2021). The number of new HIV diagnoses fluctuated for transgender women (*p*_trend_ = 0.053) and late presentation was common (ranging between 10% and 67% of new HIV diagnoses). Of the 260 transgender women, 26 (10%) disengaged from care between 2011 and 2021 (incidence rate = 1.10 per 100 person‐years, 95% confidence interval = 0.75−1.61).

**Conclusions:**

Between 2011 and 2021, less than 90% of transgender women linked to HIV care were virally suppressed. Late presentation at the time of diagnosis and disengagement from care were common. Efforts are needed to identify barriers to early HIV diagnosis and to optimize the different steps across the care continuum for transgender women.

## 1 INTRODUCTION

Transgender women are at increased risk of human immunodeficiency virus (HIV) acquisition [1−5]. Since the introduction of combination antiretroviral therapy (ART), HIV‐related morbidity and mortality have been substantially reduced. Moreover, when individuals use ART effectively and achieve viral suppression, they can no longer transmit HIV, thereby making ART one of the key HIV prevention measures [6−8]. The HIV care continuum, which illustrates the chain of events from the awareness of HIV status to achieving viral suppression, has become a pivotal instrument in monitoring the HIV epidemic and has become embedded in the Joint United Nations Programme on HIV/AIDS (UNAIDS) 95‐95‐95 testing and treatment targets for 2025 [9].

While country‐specific annual estimates on the HIV care continuum comprising the overall population of people living with HIV are common, only few countries, and none in Europe, have reported specifically on the HIV care continuum among transgender individuals [2, 10−19]. Assessing the HIV care continuum for specific key populations, like transgender women, is crucial to identify areas for improvement in HIV testing and care programmes. This is even more urgent as the few studies which have estimated the care continuum for transgender women have shown suboptimal retention in care, uptake of and adherence to ART and viral suppression [10−19].

Until now, HIV care continuum outcomes among transgender women specifically have not been evaluated in the Netherlands. Therefore, in this study, we aimed to assess the stages of the HIV care continuum among transgender women with HIV in care in the Netherlands over an 11‐year period (2011−2021). Additionally, we assessed the annual number of new HIV diagnoses, late presentation to care, as well as the rate of disengagement from care during the same period.

## 2 METHODS

In the Netherlands, HIV care is provided by 24 HIV treatment centres located throughout the Netherlands. Their services are available to all people living with HIV (including undocumented and uninsured people). Dutch treatment guidelines are followed and include rapid ART initiation [20]. Same‐day ART initiation is not country‐wide applied in the Netherlands, but rapid ART initiation is universally applied. Access to HIV care is generally low‐threshold and HIV treatment centres initiate ART rapidly after diagnosis. Initiating ART during one of the first clinic visits to an HIV treatment centre has improved future engagement in care in the Netherlands, as compared to the immediate start of ART by the provider who established the diagnosis.

The Dutch HIV Monitoring Foundation (Stichting hiv monitoring [SHM]) collects data from as many as 98% of the individuals receiving care in these centres, comprising the ATHENA (AIDS Therapy Evaluation in the Netherlands) cohort [21]. Demographic data and relevant HIV and treatment data are prospectively and continuously collected in the cohort.

### 2.1 Study design and population

For this study, we included data from transgender women with HIV from the ATHENA cohort. We annually assessed the proportion of transgender women in different stages of the HIV care continuum between 2011 and 2021. We also assessed the annual number of newly diagnosed transgender women and among those the proportion of late presenters (defined as those with either a CD4 count of <350 cells/µl or an AIDS diagnosis at the time of diagnosis, and no evidence of having acquired HIV in the 12 months before diagnosis). Transgender women who were registered at SHM and attended clinical visits between 1 January 2011 and 31 December 2021 were eligible if they self‐identified as a transgender woman and were 18 years or older at the time of HIV diagnosis.

### 2.2 Measurements

At the time of enrolment into the ATHENA cohort, the following demographic information was collected: year of birth, country of birth, sex assigned at birth, gender identity if different from sex at birth (based on self‐report) and most likely transmission route of HIV (e.g. sexual contact). Information on gender identity was collected from 2015 and if someone identified as transgender, gender identity was retrospectively corrected. Information about the date of HIV diagnosis was retrieved from the referral letter of the general practitioner or Centre for Sexual Health, from health records in the HIV treatment centre, or self‐reported if no documentation was available [21].

We used information collected by trained SHM data collectors from regular clinic visits concerning the occurrence of AIDS‐defining conditions and ART use. Lab results on peripheral blood CD4 cell count and HIV‐RNA test results were imported directly into the ATHENA database from clinical records.

### 2.3 HIV care continuum

SHM annually reports about the HIV care continuum entailing five stages [22]: (1) total number of individuals with HIV in the Netherlands; (2) individuals with HIV who have been diagnosed and linked to care; (3) individuals linked to care who are retained in care; (4) individuals retained in care receiving ART; and (5) individuals receiving ART who are virally suppressed. The first stage in the continuum also includes the estimated number of people with HIV who have not yet been diagnosed and this number is estimated using mathematical modelling strategies [22]. However, due to the limited number of transgender women in the ATHENA cohort and uncertainty in the population estimates of HIV among transgender women in the Netherlands, we were unable to obtain a reliable estimate of this first stage. Therefore, for the purpose of the current study, we restricted our analyses to stages 2 through 5 of the care continuum, using the following definitions.

#### 2.3.1 Diagnosed and linked to care

All transgender women in care in the Netherlands as of 2010 were considered linked to care. Transgender women who, in a given calendar year, were known to have died or moved to another country were excluded from our estimates from that year onwards. Additionally, we excluded transgender women who did not attend any HIV clinical visits in the 10 years prior to a given calendar year. Provided follow‐up started in 2010, transgender women were excluded from 2020 (i.e. 10 years after 2010) onwards if no further clinical visits were registered.

#### 2.3.2 Linked to and retained in care

Among those linked to care, the transgender women who attended at least one clinic visit at an HIV treatment centre or had at least one CD4 or viral load measurement in a particular year.

#### 2.3.3 On ART, not virally suppressed

Among those linked to and retained in care, the transgender women who had received ART for at least 6 months (to assess consistent ART use within a year) within a particular calendar year with an HIV‐RNA assessment ≥200 RNA per ml.

#### 2.3.4 On ART and virally suppressed

Among those on ART, the transgender women who had an undetectable HIV viral load (defined as <200 copies of viral RNA per ml) at their final visit within a given calendar year.

### 2.4 Statistical analyses

Demographic and clinical characteristics at the first clinic visit between 2011 and 2021 were described using absolute number and proportions for categorical variables and median and interquartile range (IQR) for continuous variables. Total numbers were provided, as well as numbers by period of HIV diagnosis (before 2015 vs. from 2015 onwards).

#### 2.4.1 HIV care continuum

We visualized the proportion of transgender women in each stage of the care continuum annually from 2011 to 2021. In addition, we calculated the median and IQR of the number of viral load results measured among transgender women for every calendar year between 2011 and 2021.

#### 2.4.2 New HIV diagnoses

Among all transgender women linked to care between 2011 and 2021, we assessed the annual proportion with a new HIV diagnosis. We assessed fluctuations in the number of new HIV diagnoses and the number of late diagnoses (i.e. late presenters, defined as those with either a CD4 count of <350 cells/µl or an AIDS diagnosis at the time of diagnosis, and no evidence of having acquired HIV in the 12 months before diagnosis) between 2011 and 2021 using the Cochran–Armitage test for linear trends. Among the transgender women with a new HIV diagnosis between 2011 and 2021, we assessed the median time from diagnosis until ART initiation and the median time from ART initiation until viral suppression. We also assessed the time from HIV diagnosis until ART initiation and from ART initiation until viral suppression, both before and after 2015. At that time, national and international guidelines were adapted to recommend starting ART immediately after diagnosis, and also the use of integrase inhibitors known to result in more rapid suppression of HIV increased as part of first‐line ART [23, 24].

#### 2.4.3 Disengagement from care

Among all transgender women linked to care between 2011 and 2021, we estimated the incidence rate of disengagement from care (i.e. moved to another country or no HIV clinical visit for 2 years) by dividing the number of disengaged from care by the total amount of person‐time contributed. Person‐time started from the first ever registered clinic visit in the SHM database (i.e. including visits before 2011) and ended the moment someone was registered as disengaged from care.

We carried out analyses using Stata (v17.0, StataCorp, College Station, TX, USA).

### 2.5 Ethical considerations

As of 2002, the ATHENA cohort study is managed by SHM, the institution appointed by the Dutch Ministry of Public Health, Welfare and Sport for the monitoring of people living with HIV in the Netherlands. At initiation, the ATHENA cohort study was approved by the institutional review boards of all participating centres. People entering into HIV care receive written material about participation in the cohort and are informed by their treating physician on the purpose of data collection, after which they can consent verbally or elect to opt‐out. Data from the cohort are pseudonymized before being provided to investigators and may be used for scientific purposes. A designated data protection officer safeguards compliance with the European General Data Protection Regulation [21].

### 2.6 Role of the funding source

The funder had no role in the study design, data collection, data analysis, data interpretation and writing of the report.

## 3 RESULTS

Between 1 January 2011 and 31 December 2021, 260 self‐identified transgender women attended at least one clinic visit at an HIV treatment centre in the Netherlands. Median age at the first HIV clinical visit was 34 years [IQR 27−41] and 53 (20%) were born in the Netherlands (Table 1). Median age at the time of HIV diagnosis was 28 years [IQR 24−35] and median time from HIV diagnosis to linkage to care was 18 days [IQR 6−75].

**Table 1:** Socio‐demographic and clinical characteristics at the first HIV clinical visit between 2011 and 2021 of transgender women with HIV, the Netherlands

	Total		Diagnosed up to 2015 (*n* = 191)		Diagnosed from 2015 (*n* = 69)	
	*n* ^a^	%^a^	*n* ^a^	%^a^		
**Age (in years)**						
Median [IQR]	34	[27−41]	35	[27−42]	29	[27−35]
<25	33	13%	25	13%	8	12%
25−34	107	41%	65	34%	42	61%
35−44	81	31%	68	36%	13	19%
≥45	39	15%	33	17%	6	9%
**Region of origin**						
The Netherlands	53	20%	38	20%	15	22%
Other^b^	207	80%	153	80%	54	78%
*Western Europe*	*8*	*3%*	*8*	*4%*	*0*	*0%*
*Central Europe*	*1*	*0.4%*	*0*	*0%*	*1*	*1%*
*Eastern Europa & Central Asia*	*2*	*0.8%*	*1*	*0.5%*	*1*	*1%*
*Sub‐Saharan Africa*	*6*	*2%*	*4*	*2%*	*2*	*3%*
*East Asia & Pacific*	*1*	*0.4%*	*1*	*0.5%*	*0*	*0%*
*Australia & New Zealand*	*0*	*0%*	*0*	*0%*	*0*	*0%*
*South & South‐East Asia*	*29*	*11%*	*22*	*12%*	*7*	*10%*
*North Africa & Middle East*	*10*	*4%*	*9*	*5%*	*1*	*1%*
*North America*	*0*	*0%*	*0*	*0%*	*0*	*0*
*Caribbean*	*54*	*21%*	*42*	*22%*	*12*	*17%*
*South America*	*96*	*37%*	*66*	*35%*	*30*	*43%*
**Age at HIV diagnosis**						
Median [IQR]	28	[24−35]	28	[24−35]	29	[26−34]
**Time between HIV diagnosis and linkage to care (days)**						
Median [IQR]	18	[6−75]	21	[5−28]	13	[5−28]
**Time between HIV diagnosis and entry into care >6 weeks**						
No	183	70%	128	67%	55	80%
Yes	77	30%	63	33%	14	20%
**First CD4 cell count at/after diagnosis (cells/µl)** ^c^						
Median [IQR]	370	[200−570]	340	[150−580]	415	[260−565]
<200	45	25%	35	28%	10	18%
200−499	73	40%	47	38%	26	46%
≥500	63	35%	43	34%	20	36%
**First HIV viral load at/after diagnosis (copies/ml)** ^d^						
<200	18	10%	13	11%	5	9%
≥200	160	90%	109	89%	51	91%

Abbreviations: HIV, human immunodeficiency virus; IQR, interquartile range; ml, millilitre.

^a^ Unless otherwise indicated.

^b^ Other category is detailed in italic below.

^c^ Seventy‐nine transgender women did not have a CD4 cell count within 6 months of diagnosis.

^d^ Eighty‐two transgender women did not have a viral load measurement within 6 months of diagnosis.

### 3.1 HIV care continuum

**Figure 1:**
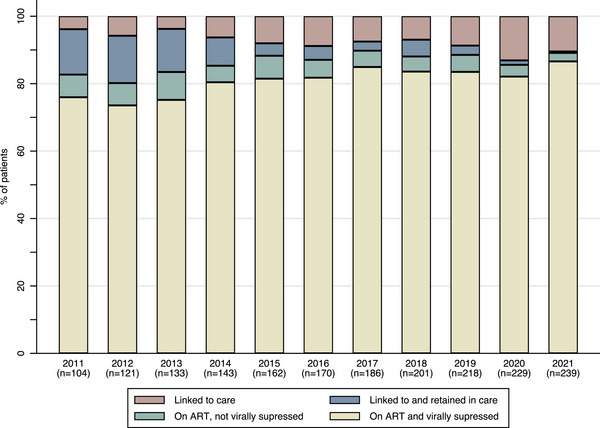
Retention in care, ART use and viral suppression among transgender women who were linked to care between 2011 and 2021, the Netherlands.

Over time, the number of transgender women linked to care increased from 104 in 2011 to 239 in 2021. The majority of transgender women, who were linked to care, were retained in care, received ART and were virally suppressed between 2011 and 2021 (Figure 1 and Table S1). However, the proportion of transgender women linked to and retained in care, on ART and virally suppressed was below 90% in all years (Table S1). Of transgender women linked to care in 2021 (i.e. all individuals linked to care since 2010, who had not died or moved to another country and had had at least one clinic visit in the previous 10 years, *n* = 239), 214 (90%) were retained in care, 213 (89%) were using ART and 207 (87%) were virally suppressed (Table S2). If ART use and viral suppression was calculated as a proportion of those retained in care instead of those linked to care, 99.5% (213 of 214) transgender women used ART and 97% (*n* = 207) transgender women were virally suppressed. Transgender women had a median of two viral load measurements in each calendar year (Table S3).

### 3.2 New HIV diagnoses

**Figure 2:**
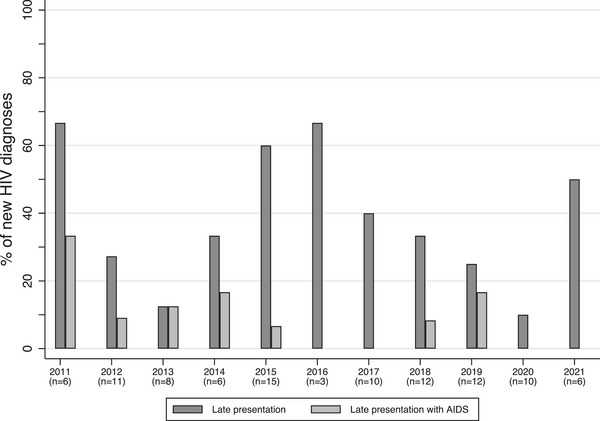
Late presentation with or without AIDS among transgender women newly diagnosed with HIV between 2011 and 2021 in the Netherlands. *The number on the x‐axis corresponds to the total number of new HIV diagnoses in that year. The bars indicate the proportion of new HIV diagnoses which were late presentations and late presentations with an AIDS diagnosis. A late diagnosis was defined as a CD4 count of <350 cells/µl or AIDS at the time of HIV diagnosis, and no evidence of having acquired HIV in the 12 months before diagnosis*.

There appeared to be a downward trend over time of new HIV diagnoses among transgender women, albeit non‐significant (*p* = 0.053), and late presentation was common (between 10% and 67% of new HIV diagnoses) (Table S4 and Figure 2). Between 0% and 33% of transgender women with a new HIV diagnosis between 2011 and 2021 presented with AIDS at the time of diagnosis (Figure 2).

The median time between HIV diagnosis and ART initiation was 40 days [IQR 19−71] for transgender women who were newly diagnosed with HIV between 2011 and 2021. Median time from ART initiation to viral suppression was 42 days [IQR 28−99]. Time until ART initiation and viral suppression became shorter from 2016 onwards. Until 2015, median time to ART initiation was 51 days [IQR 21−301] and time from ART initiation to achieving viral suppression was 51 days [IQR 30−136]. From 2016 onwards, the time from diagnosis to ART initiation was 36 days [IQR 19−63] and the time from ART initiation to viral suppression 38 days [IQR 28−81].

### 3.3 Disengagement from care

Of the total number of 260 transgender women, 26 (10%) disengaged from care in the Netherlands between 2011 and 2021. The incidence rate of disengagement from care over time was 1.10 per 100 person‐years (95% CI 0.75−1.61). A common reason for disengagement from care was moving to another country (*n* = 12/26, 46%). For 14/26 (54%) transgender women, the reason for disengagement from care was unknown. When only considering those with an unknown reason as having disengaged from care, the incidence rate of disengagement from care was lower (0.59 per 100 person‐years, 95% CI 0.35−1.00).

## 4 DISCUSSION

Using data from the nationally representative Dutch ATHENA cohort, we provide a comprehensive overview of the HIV care continuum among transgender women over a time period of 11 years. Although we found that the majority of transgender women, who were linked to care during that period, were also retained in care, on ART and virally suppressed, the proportion virally suppressed each year was less than 90%. In addition, we found that each year, among those linked to care, the proportion newly diagnosed with HIV remained stable, and late presentation among those newly diagnosed was common.

In the Netherlands, we have seen a considerable improvement over time in the proportion of men who have sex with men (MSM) who were retained in care, on ART and virally suppressed [22], while for transgender women, this proportion fluctuated over time and was substantially lower compared to MSM: 87% versus 92% of those linked to care in 2021 [22]. Nevertheless, this percentage is higher compared to nine studies from the United States of America, Brazil, Zimbabwe, South Africa and Canada that reported proportions with viral suppression between 25% and 73% among transgender women [10−19]. It is well known that transgender people face health inequalities globally, including delayed diagnoses, gender‐related medical misattribution, increased rates of cancer, cardiovascular health issues, sexually transmitted infections and mental health problems. Additionally, trans people experience frequent barriers to care, including discrimination, lack of provider knowledge, invasive questioning and long waiting lists of a lack of gender‐affirming care, affecting their trust and use of healthcare services in general. These circumstances may limit their capacity to engage with healthcare. A possible explanation for the higher proportion of transgender women who achieved viral suppression may be that health insurance in the Netherlands is mandatory, ensuring that every inhabitant has access to healthcare, including HIV care. In addition, for undocumented or uninsured persons, special financial arrangements to ensure access to healthcare (among which HIV care) are in place (https://www.hetcak.nl/). It was encouraging to find that among those transgender women newly diagnosed with HIV, the time from diagnosis to start ART and the time from start ART to viral suppression was approximately 1 month, and when in care, the majority had at least two viral load measurements per year.

Late presentation was not uncommon over time among transgender women, with the proportion of transgender women newly diagnosed with HIV who were late presenters ranging between 10% and 67% between 2011 and 2021. A lack of trust in healthcare providers and the healthcare system might explain this finding [18, 25]. Past experiences of HIV‐related stigma, being misgendered or fear of discrimination related to gender identity have previously been identified as barriers to HIV care, retention and ART adherence [10, 18, 25]. Moreover, a fear of drug−drug interactions between feminizing hormones and ART, and prioritizing of gender‐affirming care, may play a role [18, 25]. Finally, HIV care uptake and adherence may be negatively influenced by a lack of social support of peers and family members of transgender women, which was previously found to have a big impact on the uptake of care [25, 26]. Given that almost 80% of transgender women with HIV in the Netherlands were born in Latin America, the Caribbean or South‐ and South‐East Asia, tailored interventions to these specific groups may be needed to increase engagement in care.

Disengagement from care in the Netherlands was approximately 10% among transgender women. The primary reason for disengagement from care was moving to another country than the Netherlands, which may mean HIV care was transferred to an institution abroad. The reason for disengagement from care was also often unknown and hence HIV care continuation also. It is crucial that healthcare providers find ways, together with the transgender community, to ensure continued access to HIV and sexual healthcare around and after migration for transgender women. Combining HIV prevention and treatment with gender‐affirming care, preferably designed and led by members of the transgender community, may provide a safer space for transgender women and may be effective in reducing late presentation, disengagement from care and increasing HIV care retention [27, 28]. In Amsterdam, a community‐led clinic started in 2018, providing hormone support for trans people with a migration background. From 2021, the clinic started a collaboration with the Amsterdam Center for Sexual Health, providing access to free testing for sexually transmitted infections, pre‐exposure prophylaxis (PrEP) and vaccinations. The clinic aids in linkage to HIV care within one of the established HIV treatment centres, but lacks financial and logistic requirements to provide HIV treatment. In 2023, a second location opened in the city of Utrecht. However, it is relevant to note that both clinics have limited capacity due to a lack of structural funding and have extensive waiting lists to access to gender‐affirming care.

### 4.1 Strength and limitations

Our study is one of the few, and the first from Europe, that provides insight into the HIV care continuum specifically for transgender women. We used routinely collected representative national data covering more than 98% of all people with HIV and linked to care in the Netherlands, collected over an 11‐year time span, and we used these data to assess variation within the HIV care continuum over time. All previous studies focused on one time point only [10−19]. Moreover, we used clinical and laboratory data, in contrast to other studies that relied on self‐reported data [10, 11, 13−15, 18].

Our study nonetheless also has several limitations. First, we were unable to estimate the first stage of the HIV care continuum (the estimated number of persons living with HIV who remain undiagnosed) for transgender women. The number of MSM living with undiagnosed HIV in the Netherlands is estimated yearly using mathematical modelling and longitudinal data on HIV diagnoses stratified by CD4 cell count category at the time of diagnosis [22]. The limited annual number of transgender women newly diagnosed with HIV does not allow for a reliable similar estimate for this key population. Second, socio‐demographic information collected within the ATHENA cohort is limited, which precludes more detailed analysis into associated factors (e.g. socio‐economic status and drug use) for example late presentation or having a detectable viral load. Third, the number of transgender women in this study was small, giving rise to some uncertainty in the results presented. Fourth, some transgender persons might have been misclassified as cis‐gender, which may have impacted our results depending on their uptake and outcomes of care. Specifically, we only started collecting self‐reported gender identity from 2015 onwards, before 2015 this was not registered. Data pre‐2015 were retro‐actively corrected in our database for those with a clinical visit after 2015. However, this means that transgender women, who had no clinical visits since 1 January 2015 (e.g. because of emigration, loss‐to‐follow‐up or death), were not registered as transgender women. Fifth, while the ATHENA cohort covers over 98% of people with HIV in the Netherlands, it is possible that some transgender women are part of the 2% of people with HIV who did not consent to cohort participation. While we do not know if any transgender women are part of this 2%, this means that our results may not be generalizable to all transgender women with HIV in the Netherlands. Last, the time to linkage to care and time to start ART might be an overestimation when participants initiated HIV care outside of the Netherlands and did not report this during their clinic visit.

## 5 CONCLUSIONS

Over an 11‐year time period, the majority of transgender women with HIV, who were linked to care in the Netherlands, were retained in care, received ART and were virally suppressed. Nonetheless, the HIV care continuum for transgender women is suboptimal across its stages. In addition, late presentation remains common. Efforts are needed to identify barriers to early HIV diagnosis and to optimize the different steps across the care continuum for transgender women. Identifying facilitators and barriers to these aspects of HIV care and designing targeted interventions, jointly with the transgender community, will be crucial to improve HIV care retention and outcomes.

## COMPETING INTERESTS

EH received unrestricted research grants from Gilead Sciences paid to her institute. MP received unrestricted research grants and speaker's fees from Gilead Sciences, Abbvie and MSD, all of which were paid to her institute and unrelated to the current work. MSvdL served on an Advisory Board of MSD, paid to his institute and unrelated to the current work. PR has received grant support through his institution for investigator‐initiated research unrelated to the current work from Gilead Sciences, ViiV Healthcare and Merck & Co, and has served on scientific advisory boards of Gilead Sciences, ViiV Healthcare and Merck & Co, honoraria for which were paid to his institution. All other authors declare no competing interests.

## AUTHORS’ CONTRIBUTIONS

EH, MP, PR, MvdV, CD and VWJ conceptualized and designed this study. VWJ, AvS, KH and MSvdL were involved in the data management and analysis. VWJ, CD, AvS, MSvdL, KH, CW, AvVK, MdH, EJGP, MvdV, PR, MP and EH were involved with the interpretation of the data. VWJ and CD drafted the manuscript. All authors read and approved the final manuscript.

## FUNDING

The ATHENA Cohort is managed by Stichting hiv monitoring and supported by a grant from the Dutch Ministry of Health, Welfare and Sport through the Centre for Infectious Disease Control of the National Institute for Public Health and the Environment.

Statistical information or data for separate research purposes from the ATHENA cohort can be requested by submitting a research proposal to SHM (https://www.hiv‐monitoring.nl/english/research/research‐projects/). The proposal will undergo review by representatives of SHM for evaluation of scientific value, relevance of the study, design and feasibility, statistical power, and overlap with existing projects.

## Supporting information


**Supplementary Table 1**. Proportion of transgender women in each stage of the HIV care continuum, by year between 2011–2021, the Netherlands
**Supplementary Table 2**. Proportion of transgender women in each stage of the HIV care continuum in 2021, the Netherlands
**Supplementary Table 3**. Frequency of viral load testing per year for transgender women living with HIV, by year, the Netherlands
**Supplementary Table 4**. New HIV diagnoses as a proportion of transgender women living with HIV and linked to care, by year, the Netherlands

## Data Availability

ATHENA cohort data used in this study are available upon reasonable request. Requests for data access can be made to: hiv.monitoring@amsterdamumc.nl. These will be reviewed on a case‐by‐case basis, given that the data underlying this study contain sensitive information.
