# Supplementary material for: Assessing the HIV care continuum among transgender women during 11 years of follow‐up: results from the Netherlands’ ATHENA observational cohort
Source: J Int AIDS Soc. 2024 Aug 8;27(8):e26317. doi: 10.1002/jia2.26317 (PMC11310271; doi:10.1002/jia2.26317)
Supplement: Supplementary file 1 — Supplementary Table 1. Proportion of transgender women in each stage of the HIV care continuum, by year between 2011–2021, the Netherlands Supplementary Table 2. Proportion of transgender women in each stage of the HIV care continuum in 2021, the Netherlands Supplementary Table 3. Frequency of viral load testing per year for transgender women living with HIV, by year, the Netherlands Supplementary Table 4. New HIV diagnoses as a proportion of transgender women living with HIV and linked to care, by year, the Netherlands [file JIA2-27-e26317-s001.docx]

Supplementary Table 1. Proportion of transgender women in each stage of the HIV care continuum, by year between 2011-2021, the Netherlands

|  |  | |
| --- | --- | --- |
|  | **n** | **%** |
| ***2011*** |  |  |
| Linked to care, but not retained | 4 | 4% |
| Linked and retained, but not on cART | 14 | 13% |
| Linked, retained and using cART, but not virally suppressed | 7 | 7% |
| Linked, retained, cART and virally suppressed | 79 | 76% |
| ***2012*** |  |  |
| Linked to care, but not retained | 7 | 6% |
| Linked and retained, but not on cART | 17 | 14% |
| Linked, retained and using cART, but not virally suppressed | 8 | 7% |
| Linked, retained, cART and virally suppressed | 89 | 74% |
| ***2013*** |  |  |
| Linked to care, but not retained | 5 | 4% |
| Linked and retained, but not on cART | 17 | 13% |
| Linked, retained and using cART, but not virally suppressed | 11 | 8% |
| Linked, retained, cART and virally suppressed | 100 | 75% |
| ***2014*** |  |  |
| Linked to care, but not retained | 9 | 6% |
| Linked and retained, but not on cART | 12 | 8% |
| Linked, retained and using cART, but not virally suppressed | 7 | 5% |
| Linked, retained, cART and virally suppressed | 115 | 80% |
| ***2015*** |  |  |
| Linked to care, but not retained | 13 | 8% |
| Linked and retained, but not on cART | 6 | 4% |
| Linked, retained and using cART, but not virally suppressed | 11 | 7% |
| Linked, retained, cART and virally suppressed | 132 | 81% |
| ***2016*** |  |  |
| Linked to care, but not retained | 15 | 9% |
| Linked and retained, but not on cART | 7 | 4% |
| Linked, retained and using cART, but not virally suppressed | 9 | 5% |
| Linked, retained, cART and virally suppressed | 139 | 82% |
| ***2017*** |  |  |
| Linked to care, but not retained | 14 | 8% |
| Linked and retained, but not on cART | 5 | 3% |
| Linked, retained and using cART, but not virally suppressed | 9 | 5% |
| Linked, retained, cART and virally suppressed | 158 | 85% |
| ***2018*** |  |  |
| Linked to care, but not retained | 14 | 7% |
| Linked and retained, but not on cART | 10 | 5% |
| Linked, retained and using cART, but not virally suppressed | 9 | 4% |
| Linked, retained, cART and virally suppressed | 168 | 84% |
| ***2019*** |  |  |
| Linked to care, but not retained | 19 | 9% |
| Linked and retained, but not on cART | 6 | 3% |
| Linked, retained and using cART, but not virally suppressed | 11 | 5% |
| Linked, retained, cART and virally suppressed | 182 | 83% |
| ***2020*** |  |  |
| Linked to care, but not retained | 30 | 13% |
| Linked and retained, but not on cART | 3 | 1% |
| Linked, retained and using cART, but not virally suppressed | 8 | 3% |
| Linked, retained, cART and virally suppressed | 188 | 82% |
| ***2021*** |  |  |
| Linked to care, but not retained | 25 | 10% |
| Linked and retained, but not on cART | 1 | 0.4% |
| Linked, retained and using cART, but not virally suppressed | 6 | 3% |
| Linked, retained, cART and virally suppressed | 207 | 87% |

**Abbreviations**: cART, combination antiretroviral therapy

Supplementary Table 2. Proportion of transgender women in each stage of the HIV care continuum in 2021, the Netherlands

|  | **Transgender women (n=239)** | |
| --- | --- | --- |
|  | **n** | **%** |
| Linked to care | 239 | 100% |
| Linked and retained in care | 214 | 90% |
| Linked, retained and using cART | 213 | 89% |
| Linked, retained, cART and virally suppressed | 207 | 87% |

**Abbreviations**: cART, combination antiretroviral therapy

Supplementary Table 3. Frequency of viral load testing per year for transgender women living with HIV, by year, the Netherlands

|  | **Transgender women**  **(n=260)** | |
| --- | --- | --- |
|  | **Median** | **[IQR]** |
| **2011** | 2 | [2-3] |
| **2012** | 2 | [1-3] |
| **2013** | 2 | [1-3] |
| **2014** | 2 | [2-3] |
| **2015** | 2 | [2-3] |
| **2016** | 2 | [2-3] |
| **2017** | 2 | [2-2] |
| **2018** | 2 | [2-2] |
| **2019** | 2 | [2-2] |
| **2020** | 2 | [1-2] |
| **2021** | 2 | [2-2] |

**Abbreviations**: IQR, interquartile range

Supplementary Table 4. New HIV diagnoses as a proportion of transgender women living with HIV and linked to care, by year, the Netherlands

|  | **Transgender women** | | |  |
| --- | --- | --- | --- | --- |
|  | **Living with HIV & linked to care** | **New diagnoses** | **Late diagnoses^a^** | **Late presentation with AIDS** |
|  |  | *n (%)* | *n (%)* | *n (%)* |
| **2011** | 101 | 6 (6%) | 4 (67%) | 2 (33%) |
| **2012** | 114 | 11 (10%) | 3 (27%) | 1 (9%) |
| **2013** | 129 | 8 (6%) | 1 (13%) | 1 (13%) |
| **2014** | 134 | 6 (4%) | 2 (33%) | 1 (17%) |
| **2015** | 149 | 15 (10%) | 9 (60%) | 1 (7%) |
| **2016** | 156 | 3 (2%) | 2 (67%) | 0 (0%) |
| **2017** | 173 | 10 (6%) | 4 (40%) | 0 (0%) |
| **2018** | 190 | 12 (6%) | 4 (33%) | 1 (8%) |
| **2019** | 201 | 12 (6%) | 3 (25%) | 2 (17%) |
| **2020** | 206 | 10 (5%) | 1 (10%) | 0 (0%) |
| **2021** | 216 | 6 (3%) | 3 (50%) | 0 (0%) |

1. Among patients with a new HIV diagnosis. Defined as a CD4 of <350 cells/microlitre at the first CD4 measurement after diagnosis or being diagnosed with an AIDS defining illness before/at time of HIV diagnosis, and no evidence of having acquired HIV in the 12 months before diagnosis
